# Naturally Occurring Retirement Communities: Scoping Review

**DOI:** 10.2196/34577

**Published:** 2022-04-14

**Authors:** Simone Parniak, Vincent G DePaul, Clare Frymire, Samuel DePaul, Catherine Donnelly

**Affiliations:** 1 Health Services and Policy Research Institute Faculty of Health Sciences Queen's University Kingston, ON Canada; 2 School of Rehabilitation Therapy Faculty of Health Sciences Queen's University Kingston, ON Canada

**Keywords:** naturally occurring retirement communities, NORC, NORC supportive service programs, aging in place, older adults, scoping review

## Abstract

**Background:**

As Canada’s population ages, there is a need to explore community-based solutions to support older adults. Naturally occurring retirement communities (NORCs), defined in 1986 as buildings or areas not specifically designed for, but which attract, older adults and associated NORC supportive service programs (NORC-SSPs) have been described as potential resources to support aging in place. Though the body of literature on NORCs has been growing since the 1980s, no synthesis of this work has been conducted to date.

**Objective:**

The goal of this scoping review is to highlight the current state of NORC literature to inform future research and offer a summarized description of NORCs and how they have supported, and can support, older adults to age in place.

**Methods:**

Using a published framework, a scoping review was conducted by searching 13 databases from earliest date of coverage to January 2022. We included English peer- and non–peer-reviewed scholarly journal publications that described, critiqued, reflected on, or researched NORCs. Aging-in-place literature with little to no mention of NORCs was excluded, as were studies that recruited participants from NORCs but did not connect findings to the setting. A qualitative content analysis of the literature was conducted, guided by a conceptual framework, to examine the promise of NORC programs to promote aging in place.

**Results:**

From 787 publications, we included 64 (8.1%) articles. All publications were North American, and nearly half used a descriptive research approach (31/64, 48%). A little more than half provided a specific definition of a NORC (33/64, 52%); of these, 13 (39%) used the 1986 definition; yet, there were discrepancies in the defined proportions of older adults that constitute a NORC (eg, 40% or 50%). Of the 64 articles, 6 (9%) described processes for identifying NORCs and 39 (61%) specifically described NORC-SSPs and included both external partnerships with organizations for service delivery (33/39, 85%) and internal resources such as staff, volunteers, or neighbors. Identified key components of a NORC-SSP included activities fostering social relationships (25/64, 39%) and access to resources and services (26/64, 41%). Sustainability and funding of NORC-SSPs were described (27/64, 42%), particularly as challenges to success. Initial outcomes, including self-efficacy (6/64, 9%) and increased access to social and health supports (14/64, 22%) were cited; however, long-term outcomes were lacking.

**Conclusions:**

This review synthesizes the NORC literature to date and demonstrates that NORC-SSPs have potential as an alternative model of supporting aging in place. Longitudinal research exploring the impacts of both NORCs and NORC-SSPs on older adult health and well-being is recommended. Future research should also explore ways to improve the sustainability of NORC-SSPs.

## Introduction

### Background

Supporting older adults to age in their communities has been a focus of Canadian aging strategies and policies [1,2]; however, Canada continues to fall short in developing community-based solutions that are designed for, and by, older adults. The COVID-19 pandemic has highlighted not only the challenges and risks of long-term care, but also the critical need to examine alternative community housing models. Hunt and Gunter-Hunt [3] first coined the term naturally occurring retirement communities (NORCs) in 1986, defining them as “a housing development that is not planned or designed for older people, but which over time comes to house largely older people.” With time, the body of research on NORCs has grown to include NORC supportive service programs (NORC-SSPs): initiatives that bring older adults living in NORCs and health and community supports together to offer programs and activities to foster aging in place.

### Benefits of Reviewing NORC Literature

NORCs have been described by Kloseck et al [4] as “untapped resources to enable optimal aging at home” because they offer social-relational connections and build on the strengths of communities. Since the initial paper by Hunt and Gunter-Hunt [3] defining NORCs >30 years ago, there has been a growing body of literature on NORCs and NORC-SSPs; specifically in the last year, 3 review papers have conducted broad explorations of aging-in-place models, including NORC programs, from different perspectives [5-7]. Mahmood et al [5] described key barriers and challenges of NORC-SSPs as well as cohousing and village models within the domains of the age-friendly communities framework. Hou and Cao [6] conducted a systematic review of NORCs, cohousing, and university-based retirement community literature to explore the push-and-pull factors of migration. Chum et al [7] conducted a scoping review to explore models that included NORCs, congregate housing and cohousing, sheltered housing, and continuing care retirement communities for the purpose of identifying themes across models that support aging in place. These well-designed reviews offer further insight into NORCs and NORC programs; however, no in-depth synthesis of NORC literature has been conducted to date. A review of this literature would offer several benefits. First, a review would highlight the current state of the research and identify gaps that could guide researchers in advancing the evidence related to NORCs and NORC-SSPs. Second, a review would document and describe the different variations of NORCs and NORC-SSPs along with the methods used to identify NORCs. Third, a review would identify how and in what ways NORCs can be, and have been, used to support older adults in their community and document the benefits of NORCs to the health and well-being of individuals and to communities as a collective. Finally, a review can offer critical data that could be used to advocate for further support of NORCs. The objective of this paper is to describe the state of the literature on NORCs.

## Methods

### Approach

A scoping review was considered the appropriate approach, given the fact that no previous review of the literature had been conducted. Furthermore, our aim is to capture the full breadth of the literature, bringing all scholarly work on NORCs together, as opposed to analyzing the methodological quality of the existing evidence [8].

A scoping review as outlined by Arksey and O’Malley [9] and updated by Levac et al [10] was conducted. The scoping review followed the 5-step process proposed by Arksey and O’Malley [9]: (1) identifying the initial research question; (2) identifying relevant studies; (3) selecting the studies; (4) charting the data; and (5) collating, summarizing, and reporting the findings [9]. A sixth step, consulting with stakeholders, is considered optional and was not included in this review.

### Identifying the Research Question

As per the recommendations of Levac et al [10], we kept the research question broad but with “a clearly articulated scope of inquiry.” The research question included clearly defining key concepts (NORCs), the population of focus (older adults), and the outcomes (support). Thus, the following research question was developed to guide the search: How and in what ways do NORCs support older adults to remain living at home in their communities?

We articulated three subquestions to help guide the data extraction:

1. What methods are used in the literature to identify NORCs?
2. What mechanisms or resources are in place in NORCs and how are these provided (delivered)?
3. What outcomes are used to determine the benefits of NORCs?

### Identifying Relevant Studies

A professional health sciences librarian (Paola Durando) performed the scoping review search in July 2020. A subsequent search was conducted by the authors in January 2022. To conduct a comprehensive search within NORC literature, the only search term used was naturally occurring retirement communit*. Expanders to include equivalent subjects and related words were used in some databases. Databases were searched from their earliest data of coverage through January 2022. The following databases were searched: CINAHL, Ovid MEDLINE, HealthSTAR, Embase, APA PsycINFO, Allied and Complementary Medicine Database, JBI Evidence-Based Practice Resources, REHABDATA, Sociofile, Education Source, Education Resources Information Center, Urban Planning, and the Cochrane Library. In addition, the 3 aforementioned review papers [5-7] were examined and cross-referenced to handpick additional references that were not identified in our search.

### Study Selection

Using the key search term naturally occurring retirement communit*, 787 articles were identified from across the selected databases. These articles were imported and screened for study selection using Covidence screening software (Veritas Health Innovation Ltd). Many of these articles (343/787, 43.8%) were duplicates from 92% (12/13) of the databases and removed before screening. A search of REHABDATA yielded zero results. Only duplicates were found in Google and Google Scholar searches. An initial title and abstract screening was conducted, and the inclusion and exclusion criteria were applied to guide final study selection. All titles and abstracts were screened by 2 members of the research team (SD and CF). The inclusion and exclusion criteria are specified in Textbox 1. Any discrepancies were reviewed by a third member (SP) and discussed with the initial reviewer until consensus was reached.

**Figure 1:**
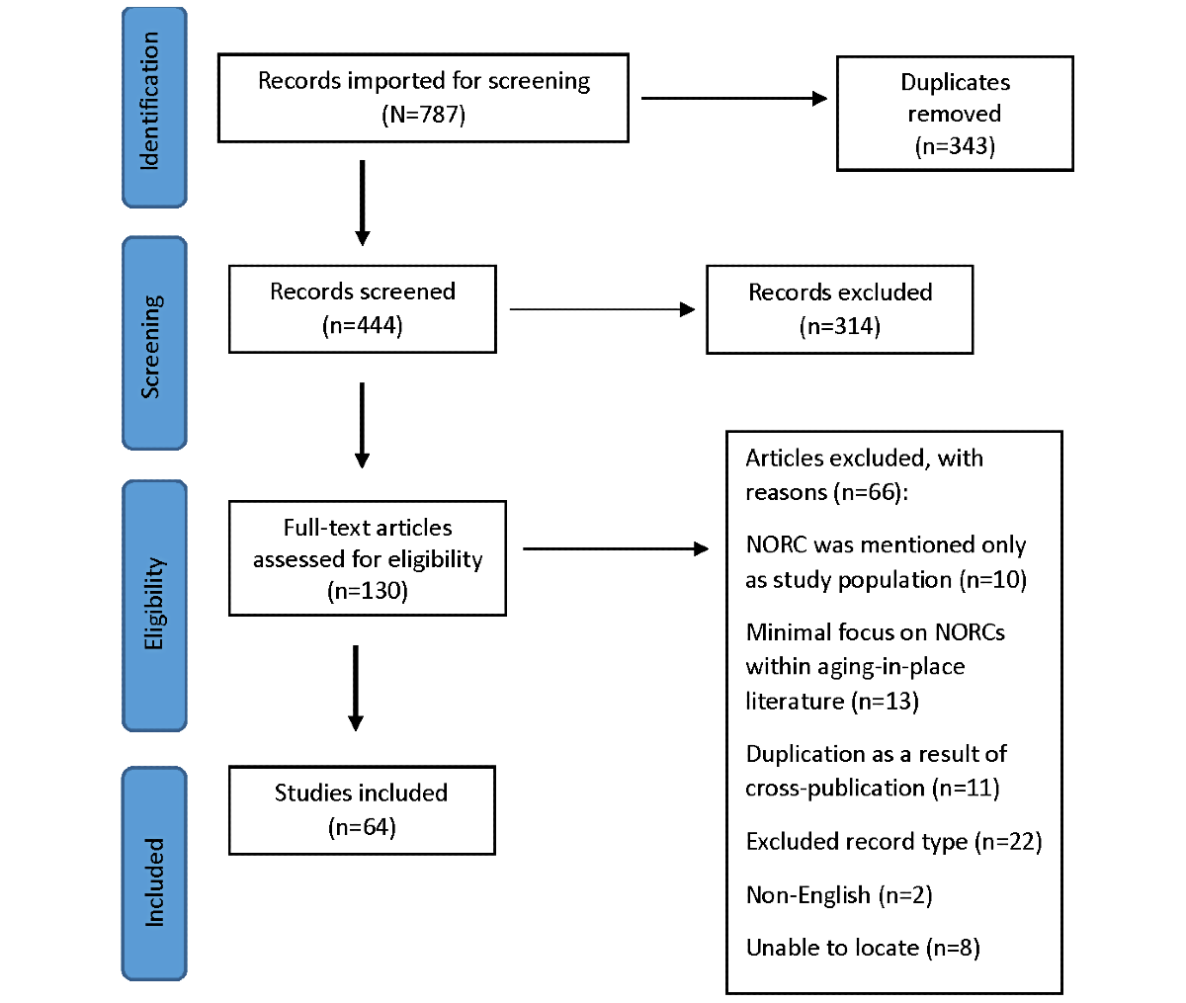
PRISMA (Preferred Reporting Items for Systematic Reviews and Meta-Analyses) flow chart. NORC: naturally occurring retirement community.

Articles identified in the abstract and title screening as relevant for a full-text review (130/784, 16.6%) were reviewed by 2 members of the research team (SD and CF) by applying the inclusion and exclusion criteria. Any discrepancies were reviewed by a third member of the research team (SP) and discussions were held until consensus was reached. Article selection followed the PRISMA (Preferred Reporting Items for Systematic Reviews and Meta-Analyses) statement, and the study process is described in Figure 1 [11]. During the full-text screening, a subset of articles (10/130, 7.7%) was excluded because of the lack of direct focus on NORCs. These articles primarily consisted of studies that sampled from different populations of older adults, some of whom lived in NORCs. The primary objectives of these studies were not to understand or demonstrate the impact of living in NORCs, and there was no specific reference to NORCs in the results or discussion. Other articles that fell into this category were those that mentioned NORCs in passing within larger discussions of aging in place (13/130, 10%). We chose to exclude both these categories of studies from the final extraction because they did not directly contribute to answering the research question. In addition, any article that was not from a scholarly source was excluded (22/130, 16.9%). After full-text review, of the 130 studies, 64 (49.2%) were selected for extraction.

**Inclusion and exclusion criteria.**

**Inclusion criteria**

- English language
- Scholarly sources, including peer- and non–peer-reviewed journals
- Subject matter
  - Descriptions
  - Critiques
  - Reflections on naturally occurring retirement communities
  - Research in naturally occurring retirement communities

**Exclusion criteria**

- Non-English
- Article types
  - Book chapters
  - Dissertations
  - Conference abstracts
  - Reports
  - Magazine or newspaper editorials
- Subject matter
  - Aging-in-place literature with little or no mention of naturally occurring retirement communities
  - Research that sampled from naturally occurring retirement communities but did not connect results to setting

### Charting the Data

During the study selection phase, the authors developed a data charting table. Variables included author, date, location, study design, and NORC definition used. Data extraction was informed by the conceptual framework developed by Greenfield et al [12], which examines the potential of NORC programs to promote aging in place. This framework identifies the different elements of NORCs as well as the outcomes of NORCs at the individual, community, and organization levels. The conceptual framework is aligned with the studies’ research questions and structures the presentation of the results.

As per the recommendations of Levac et al [10], 2 authors (SD and CF) independently extracted data from 8% (5/64) of the articles before convening to determine the consistency of their approach. Once consensus was reached, the authors proceeded with the full data extraction. Once data were extracted, the authors conducted a qualitative content analysis of the final articles (n=64), guided by the framework developed by Greenfield et al [12].

### Ethics Approval

As this is a scoping review, ethics approval was not required.

## Results

### Overview

Of the 64 articles included in this scoping review, 60 (94%) were written in a US context, with the remaining 4 (6%) written from Canadian perspectives [4,13,14,15]. The articles were published between 1985 and 2021; Table 1 shows the breakdown of the published articles by decade. Of the 64 articles, 25 (39%) presented information about NORCs, whereas the remaining 39 (61%) specifically looked at NORC-based programs. Authors of nearly half (31/64, 48%) of the articles used a descriptive research approach; of these, most were cross-sectional (25/31, 81%). Of the 64 articles, 8 (13%) [3,14,16-21] presented general descriptions or overviews of NORC or NORC-SSP concepts. Articles presented findings from, or described, a single NORC or NORC program (25/64, 39%) or ≥2 NORCs (24/64, 38%). Other articles presented descriptions of approaches to identifying NORCs [22,23] or descriptions of frameworks and tools to characterize NORCs or NORC programs [12,24,25]. In 11% (7/64) of the articles, authors compared examples of NORCs or NORC programs to other aging-in-place concepts, including the village model [26-29], campus-affiliated retirement communities [29], or new urbanism [30], and in 3% (2/64) of the articles, authors compared the concepts of horizontal and vertical NORCs [31,32]. Authors conducted studies comparing two groups within NORCs in which one receives an intervention and the other does not in 5% (3/64) of the included articles [33-35]. Methods used to conduct research in NORCs typically included surveys, interviews, focus group discussions, and observational methods.

**Table 1:** Decade of publication.

Years	Number of articles
1980-1989	1
1990-1999	1
2000-2009	20
2010-2019	42

### How Are NORCs Identified?

#### Definition of NORCs

Of the 64 articles, 33 (52%) provided a specific definition of NORCs. Authors of 20% (13/64) of the articles cited the 1986 definition proposed by Hunt and Gunter-Hunt [3]. Some authors (12/64, 19%) went further to define NORCs by including the percentage or proportion of the community and the age of its residents [17,21,24,29,31-33,36-40]. The range of inclusion criteria cited in the literature is demonstrated in Table 2.

**Table 2:** Summary of naturally occurring retirement community inclusion criteria.

Percentage and age	Citation	Studies in which the citations were found
50% are older adults	Hunt and Gunter-Hunt [3]	[17,21,29]
40%-50% of the population is aged >60 years	New York State definition [41]	[37]
50% of the residents aged >65 years	Hunt [42]	[24,33]
50% of the residents should be aged >60 years	Hunt and Ross [43]	[31,37,40]
50% of the residents are aged ≥60 years	Lanspery [44]	[38]
≥50% of the population is aged at least 50 years	Hunt [30]	[39]

A subset of articles focused specifically on NORC-SSPs: authors cited definitions from the study by Vladeck [45] that focus on bringing partnerships together to deliver services to concentrated areas of older adults [21,26,31,46]. Although most of the articles written about NORCs were indeed describing a geographical area inhabited by a large proportion of older adults, some authors referred to NORCs as communities with purposeful programs, services, and activities to assist the older adult residents but did not differentiate these as NORC-SSPs or NORC programs. For the purpose of this review, we consider these articles as part of the body of both NORC and NORC-SSP literature.

All articles presented NORCs or NORC programs within North America. When locations were specified, NORCs and NORC-SSPs were described in Wisconsin (5/64, 8%), New York State (18/64, 28%), Florida (3/64, 5%), Georgia (4/64, 6%), Missouri (4/64, 6%), Maryland (3/64, 5%), California (1/64, 2%), New Jersey (2/64, 3%), and Oregon (1/64, 2%) in the United States, and Ontario in Canada (4/64, 6%).

Aurand et al [47] explored neighborhood NORCs in Tallahassee, Florida, and described differences in rural, rural development, suburban, single-family, multifamily residential, urban commercial, and urban mixed residential neighborhoods, finding that even the most urban neighborhoods in a midsized city may lack convenient access to amenities that support aging in place. Hunt and Gunter-Hunt [3] explained in their pivotal 1986 article that NORCs vary greatly and may range in size from a single apartment building to an entire neighborhood. As NORCs are *naturally* occurring, the literature represented a range of NORCs: some authors described vertical NORCs or those in apartment buildings with or without programs (16/64, 25%), whereas others described horizontal or neighborhood NORCs with or without programs (24/64, 38%). In addition, authors compared vertical and horizontal NORCs and NORC programs (5/64, 8%) [31,32,46,48,49].

#### Methods Used to Identify NORCs

Among the 64 included publications, the authors of 6 (9%) articles about NORCs described identification processes. The purpose of 33% (2/6) of these articles was to present the process of identifying NORCs [22,23]; in both, authors used US Census data to identify areas with large proportions of older adult residents. Of the remaining 4 articles, 3 (75%) presented similar processes to identifying NORCs as part of descriptive case studies [25,47,50]. The exploration of older adult migrants in rural areas by Hunt et al [51] used a different method of identification, choosing to compile a list of rural Wisconsin NORCs by surveying key informants of local aging initiatives or through the University of Wisconsin. Key informants were asked to identify “rural areas or towns with a population of less than 10,000 residents in your country that have attracted numerous older people (aged 65+) as either permanent or seasonal residents” [51].

Among the 39 NORC-SSP articles, in 9 (23%), authors described some methods for identifying the NORCs they described: an article presented a 1991 analysis of housing occupancy in New York State to identify potential NORCs [45]. The remaining articles presented relationships as the way of identifying NORCs and NORC programs. Anetzberger [52] described a Cleveland, Ohio, NORC collectively mobilizing to secure a federal grant, whereas in the article by Altman [37] about a New York, New York, NORC-SSP, the author describes the NORC’s connection to the UJA Federation of New York, a local agency network that has played a critical role in the development of NORC programs in New York State [36]. Similar connections to local organizations were presented for other NORCs in Georgia [37,53], New York State [21,39,54,55,56], and California [31].

### What Are the Mechanisms and Resources in Place in NORCs?

#### Overview

This section of the results builds on the conceptual framework developed by Greenfield et al [12] to examine the promise of NORC programs and the village model to promote aging in place. A summary of all results related to this framework can be found in Table 3.

In 72% (46/64) of the articles, authors described resources within NORCs, which are categorized as internal and external resources. Most of the findings presented in this section refer to articles written about NORC-SSPs, rather than geographic NORCs, because NORC-SSPs include programs of some kind.

**Table 3:** Summary of key findings from the included articles (N=64)^a^.

Domain and categories				Articles, n (%)		Examples
**Resources**						
	External resources: partnerships with external service delivery and planning organizations [12]		33 (52)		[4,12-17,19-21,24,27-29,31,32,36-40,45,46,48,50,52,53-61]	
	Internal resources: staff, volunteers, and organizational mission of program		21 (33)		[4,12,15,16,21,26-28,31,36-40,45-50,52-57,59-63]	
**Activities and services**						
	Civic engagement and empowerment activities		16 (25)		[4,15-17,21,31,36,37,39,46,50,52,55,56,59,62,63]	
	Social relationship–building activities		25 (39)		[12,15-17,21,27-29,31,32,37-40,46,50,52,56,58,59,62-66]	
	Services to enhance access to resources		26 (41)		[16,17,21,27-29,31,32,36,38-40,46-50,52,53-56,58,61-63,65,66]	
**Initial outcomes**						
	Participants’ greater self-efficacy, collective efficacy, and sense of community		11 (17)		[15-17,21,32,50,52,53,62,67,68]	
	Participants’ greater social support and reduced isolation		10 (16)		[15,19,33,48-50,52,53,61,62]	
	Participants’ greater ability to access support and reduced unmet needs		14 (22)		[15-17,21,29,40,50,52,53,57,59,61,69]	
**Intermediate outcomes**						
	Individual-level, community-level, and organization-level benefits		6 (9)		[26,31,33,35,50,57]	
**Long-term goal**						
	Aging in place		1 (2)		[50]	
**Other domains: funding and sustainability**						
	Philanthropic and organizational grants		11 (17)		[21,26,29,31,33,36,39,53,55,57,63]	
	Government funding		17 (27)		[4,17,21,27,29,31-33,37,45,50,55,56,60,61,63,65]	
	Co-op board funding		1 (2)		[37]	
	Membership fees		6 (9)		[21,27,31,33,39,63]	
	Small donations and annual funding		4 (6)		[31,39,60,63]	

^a^From the conceptual framework developed by Greenfield et al [12].

#### External Resources

Greenfield et al [12] define external resources as “partnerships with external service delivery and planning entities.” In 52% (33/64) of the articles, authors described external resources, demonstrating their importance to the success of the NORC programs. Of the 64 articles, 19 (33%) presented external partnerships with health-related service organizations; Vladeck and Segel [45] describe New York, New York, NORC programs as having a health partner that is typically a home care agency, local hospital, nursing home, or combination of agencies that connect into the NORC to provide services. Authors reported health partners from service areas that included nonspecified health services [17,19,24,27,45], home care services [17], primary care physicians [57,58], nursing [4,21,36,46,53-57], occupational therapy [50,53], social work and counseling [21,24,36,46,50,53-57], pharmacy [57], and hospital-specified services [29,57].

An important external partner described in the literature was the landlord or property manager [15,32,36,52,55,56]. In some cases, the relationship was described as a financial partnership [37,52]. Altman [36] states that the landlords’ “financial participation is crucial, not only because the funds are needed to support the program’s operation but also because they become invested in a critical way in helping make the program a success. We found that having paid for a seat at the table, the housing company becomes engaged in both the planning and implementation of programs and feels more comfortable turning to the supportive service program for help when it identifies a resident in trouble.” In other contexts, the role of the landlord had less focus on financial contributions and more on in-kind contributions, including the use and maintenance of space for programs and activities [15,32].

Other external partners described in the literature included transportation agencies [17,24], churches [36], university partners (for research and student training) [21,50,59], and community agencies providing social activities [24,36,50]. In some cases, community agencies hosted outreach programs at their own facilities [48,46].

#### Internal Resources

Greenfield et al [12] describe internal resources as staff, volunteers, and organizational mission of the NORC program. Of the 64 articles, 21 (33%) presented the role of hired staff in running and supporting the NORC programs. Anetzberger [52] reports that the Community Options NORC-SSP employs resource coordinators who “help older residents on site to identify needs and then access or develop services or activities to address these needs.” A St Louis, Missouri, NORC-SSP had an entire team dedicated to supporting the program, including an activities coordinator, an outreach coordinator, a support services coordinator, a research and leadership development liaison, and a manager to oversee operations and administration [50]. This team worked to strengthen and develop the external community partnerships supporting the neighborhood.

Authors referred to the role of volunteers in running NORC programs in 22% (14/64) of the articles [4,12,15,21,28,31,36,45,48,49,56,59,60,62]. Opinions regarding the value of volunteers were sometimes mixed. Greenfield and Frantz [60] found in interviews with NORC program leaders that some felt that volunteer programs require significant staff oversight and volunteers were perceived as less accountable than staff, whereas others felt that without volunteers they would not be able to provide a sufficient number of programs to their membership. Authors described volunteers as both community members outside of the NORC and NORC-SSP participants themselves. Enguidanos et al [31] described a NORC program in metropolitan Los Angeles, California, that has a robust volunteer program, consisting primarily of older adults who were members of the NORC. Although there were struggles in recruiting and retaining volunteers (largely in part because of scheduling and long-term commitment issues), the volunteers provided 3141 hours of support to the NORC-SSP in a little more than 3 years, consisting primarily of individual supportive services such as peer counseling and friendly visits. Interestingly, the examination by Greenfield et al [28] of volunteering in age-friendly supportive service initiatives, including NORC-SSPs, found that programs with larger numbers of paid staff were associated with lower levels of older adult volunteer participation, but this had no impact on community volunteer participation.

Other internal resources mentioned in the literature included the role of neighbors and other NORC residents in supporting each other [4,21,26,49,55,56,62], whether through participation in formal advisory groups of the NORC programs or in providing peer-to-peer support to other members. The examination by Greenfield [49] of the role of neighbors’ support in NORCs revealed that NORC-SSP members felt that neighbors were valuable for information sharing and for informal network expansion but that participants sometimes valued more the services and support provided by staff and external community partnerships.

#### Activities and Services

NORC-SSPs typically consist of activities and services to support older adults to age well in their communities. Greenfield et al [12] categorize activities and services into three broad categories: civic engagement and empowerment activities, social relationship–building activities, and services to enhance access to resources.

##### Civic Engagement and Empowerment Activities

NORC-program members were described as actively engaged in 25% (16/64) of the articles. In some articles, empowerment and engagement was described as members taking on volunteer roles within the program [31,36,39]. Elbert and Neufeld [50] described a method of community building in which groups of neighbors developed “Resident Councils.” These councils met monthly to learn about available resources from each other and identify opportunities to work together toward common goals. Enguidanos et al [31] reported that although older adult engagement is important in the NORC-program model, it was difficult at early stages of development in 2 Los Angeles, California, NORCs to get older adult members to take on major roles and responsibilities.

##### Social Relationship–Building Activities

Authors described activities to build social relationships in 39% (25/64) of the articles. Examples included coffee hours [31, 62, 64], craft and hobby groups [15, 31, 56, 59], book clubs [40, 46], friendly visits [27, 28, 37, 58], day trips and outings [21, 31, 32,46, 50, 52, 62], congregate meals [15, 16, 21, 27, 31, 39, 52, 62, 65, 66], nutrition programming [17, 29, 40, 65], exercise classes [15, 17, 21, 29, 31, 37, 38, 56, 59, 62, 65], and guest speakers and education classes [17, 21, 29, 31, 38-40, 46, 50, 62, 63, 65, 66].

##### Services to Enhance Access to Resources

Of the 64 articles, 26 (41%) presented NORC-SSP offerings, including services that enhanced access to resources. In 16% (10/64) of the articles, authors described the offering of educational classes and guest speakers to the NORC-program memberships, whereas 16% (10/64) presented case management [17,29,32,36,38,48-50,63,65], 13% (8/64) presented increased access to health assessments and screenings [38-40,53,56,58,61,65], and 17% (11/64) presented the provision of services referral [16,21,27,28,36,50,52,53,56,58,63].

#### Funding and Sustainability

Authors discussed funding and sustainability in 42% (27/64) of the included articles. Among these 27 articles, in 3 (11%), authors broadly discussed the importance of funding NORC programs [14,20,38], whereas the remaining articles described examples of various sources of funding used to support NORCs and NORC programs. Funding came from philanthropic and organizational grants (11/64, 17%), government funding (17/64, 27%), co-op board funding (1/64, 2%), membership fees (6/64, 9%), and small donations and annual fundraising (4/64, 6%).

Although many funding sources were acknowledged, authors described challenges in maintaining the sustainability of NORC programs in some contexts. In the exploration by Greenfield and Frantz [60] of sustainability processes among NORC-SSPs, the authors found that respondents identified the diversification of funding sources as a key sustainability strategy. Although many authors referred to government funding, a respondent in the study by Greenfield and Frantz [60] explained as follows: “So much of our budget relies on the generosity of the state, and we consider them a partner. But every year it is a struggle to convince legislators that this is a worthy program to put resources toward.” Other articles reported that NORC-SSPs diversified funding by linking sustainability success with private-public partnerships [31,45,50].

### What Are the Outcomes of NORCs?

#### Overview

The articles included in this review presented a range of quantitative outcomes as well as anecdotal descriptions of the influence of NORCS on aging in place. Although the scoping review did not grade the level of the evidence presented in the studies, it is clear that robust outcome studies have not been completed; as a result, this section reports on outcomes that *show promise* for the NORC-program concept. Greenfield et al [12] organize outcomes into three categories: initial outcomes, intermediate outcomes, and long-term goals.

#### Initial Outcomes

Initial outcomes of participating in NORC programs can be grouped into three subcategories: (1) self-reported self-efficacy, collective efficacy, and greater sense of community; (2) self-reported increased social support and reduced isolation; and (3) self-reported access to support and reduced unmet needs.

NORC-program participants reported increased self-efficacy (6/64, 9%) [15,16,21,52,53,67], collective efficacy (2/64, 3%) [16,67], and greater sense of community (7/64, 11%) [17,32,50,52,53,62,68] in association with participation in the development of the NORC program or participation within the program itself.

NORC-SSP members reported increased social support and reduced isolation (10/64, 16%) through their participation in NORC programs. Increased social supports came from interactions with NORC-program staff, participation in service programs, and their increased interactions with neighbors and friends as a result of membership in the program.

NORC-program participants reported access to support and reduced unmet needs (14/64, 22%), primarily through the program’s function of providing increased access to services and information, including providing referrals, screenings, and educational workshops.

#### Intermediate Outcomes

Greenfield et al [12] posit that the initial outcomes associated with participation in NORC programs lead to other individual-level benefits, including better physical health and psychosocial well-being.

Although the literature connects initial outcomes, including increased social connections and self-efficacy, to participation in NORCs and NORC programs, only a few studies were longitudinal in design (6/64, 9%), making it difficult to identify intermediate outcomes. Those that were longitudinal in nature had mixed results: a longitudinal 5-year program evaluation of a single NORC by Elbert and Neufeld [50] found that participants self-reported improvements or maintenance of their health over time, whereas in their 2.6-year evaluation, Cohen-Mansfield et al [33] found that there were no changes in physical health when comparing members with nonmembers, although members felt that participation had improved their social life and a little more than half reported feeling less isolated since becoming a member.

Greenfield et al [12] include community-level and organization-level benefits within intermediate outcomes. Indeed, the literature supports that participation in NORC programs leads to increased linkages between partners and community [4,21,36,39,40,53,57]. Benefits at the organizational level (eg, program sustainability) are less clear; explorations of sustainability in NORC-SSPs [60,63] highlighted the complexity of maintaining a program, most notably securing ongoing funding.

#### Long-term Goal: Aging in Place

The framework developed by Greenfield et al [12] presents the notion that all initial and intermediate outcomes work to support a long-term goal of the NORC program in facilitating aging in place. There is a consensus in this body of research that (1) North American older adults prefer to age at home and (2) additional supports are required for older adults to age in place successfully. By its nature, a NORC is a naturally existing high-density area of older adults, which makes it a natural fit for older adult–focused programs and services, otherwise known as the creation of a NORC-SSP. However, insufficient data are presented in the literature to provide evidence that participation in NORCs and NORC-SSPs leads to an increase in the ability to age in place. An article by Elbert and Neufeld [50] found that NORC members moved to long-term care homes 45% less than nonmembers; indeed, in the 10% of the population who had died in their homes, the average individual was aged 90 years, suggesting that participation in a NORC was linked to increased ability to age in place. This was the only article in the review demonstrating a link between an increased stay at home and the existence of a NORC.

## Discussion

### Principal Findings

This is the first synthesis of the literature on NORCs and provides an important examination of how NORCS are described and the potential benefit of NORCs to older adults and communities. Since the 1980s, the body of literature around NORCs and NORC-SSPs has grown, as has the spread of the programs themselves. This scoping review yielded articles (n=64) that described NORCs and NORC programs across North America. Of the included articles, 94% (60/64) were written focusing on a US context. There is a notable absence of international perspectives in this body of research. However, we know that there is significant work looking at aging in the community that is being conducted in other countries; for example, cohousing work in the Netherlands [70,71] and aging-in-place research out of Japan [72]. This suggests that perhaps NORC is not a globally standardized term for describing neighborhoods or communities with high proportions of older adults living in them, and it will be important to examine whether concepts such as NORCs are described and understood in international contexts.

It is clear that, even in the similar North America context, there is a lack of consensus as to what specifically constitutes a NORC. Although the definition proposed by Hunt and Gunter-Hunt [3] was used by most authors, there were still significant discrepancies in terms of the age of NORC members and their proportions. It is also noteworthy that only a few (6/64, 9%) of the articles described methods for identifying NORCs. This lack of standardized method for the purpose of NORC identification makes it difficult to compare among and across NORCs and may also explain why most of the literature focuses on North America. NORCs very well could exist worldwide but have not been identified because of a lack of existing methods and terminology.

The literature highlighted the importance of both external resources such as partnerships and internal resources such as staff and volunteers as being key to the success of a NORC-SSP. Multiple partners are important to the successful functioning of a program; notably, Blumberg et al [53] describe >30 partnerships involved in an Atlanta, Georgia, affordable public housing NORC program, including connections to farmers’ markets, the university’s occupational therapy program, Medicare, and access to food stamps. Further research could explore partners’ roles and experiences engaging in NORC programs to provide better understanding of how partnerships and networks develop over time and contribute to the sustainability of a NORC program.

Authors also described activities and services of NORC programs in detail and looked at both social programs and service delivery. These 2 categories of activities are cited in the conceptual framework developed by Greenfield et al [12] as critical to reducing social isolation and enhancing access to supports and in turn addressing gaps in unmet needs within community-dwelling older adult populations. The study by Greenfield et al [12] has also cited the critical importance of civic engagement and empowerment activities to enhance older adults’ perceptions of both self- and collective efficacy, leading to both individual- and community-level benefits. However, only a few (16/64, 25%) articles presented older adult participants in leadership roles in the operation of NORC programs, characterized through volunteer roles, sitting on decision-making councils, and other such roles to drive development of their NORC program.

The literature included in this review also highlights the complexities around the funding and sustainability of NORC programs. A variety of means were used to fund NORC programs, including philanthropic and organizational grants, government funding, co-op board funding, membership fees, and annual fundraising. No single method seemed to be more sustainable than others, and as Greenfield and Frantz [60] reported, it seems that the key to success is to diversify funding sources. Authors who described funding tended to cite philanthropic and organizational grants (11/64, 17%; and 10/61, 16%, respectively) and government funds (17/64, 27%) as ways in which NORC programs were funded. The authors of this review would be interested to learn more about the potential for NORC programs to explore less traditional funding models that were described, including what sustainability might look like for a NORC program that adopts a social enterprise model, continually self-generating funds for operations rather than relying on more traditional grants, which can be less predictable.

Regarding the impacts of NORCS, interestingly, health and well-being outcomes were reported primarily for NORC programs, aligning well with the conceptual framework developed by Greenfield et al [12]; however, the authors of this review would be interested to gain more understanding as to whether simply living in a geographic region described as a NORC has positive impacts on older adult health and well-being or it is the leveraging of resources and supports to develop a NORC program that has the positive impact on participants.

### Implications for Research and Practice

On the basis of the findings from the literature, NORCs show great promise in initial outcomes that benefit the health and well-being of older adult participants. Although older adults in NORC programs demonstrated increased self- and collective efficacy and greater sense of community, increased social supports and reduced isolation, and self-reported access to support and reduced unmet needs, the research is largely descriptive. Although the purpose of this review was not to weigh the levels of evidence, it is clear that more robust study designs are needed. There are also significant gaps in the literature when looking at intermediate and long-term outcomes. Indeed, only a few articles (6/64, 9%) were longitudinal in nature, spanning a maximum of 5 years in study duration. Further research into NORC programs should look longitudinally at health and well-being outcomes to determine the long-term impacts of participating in a NORC program, including whether participation leads to an increased ability for an older adult to age in place. Longitudinal work should also explore organization-level benefits, including the journey of a NORC program to operational sustainability.

There are clear challenges in conducting community-based research, and traditional randomized controlled trials may not be feasible to examine NORC outcomes. Other community models to support aging, including the village model in the United States [73,74] and cohousing models in Europe [70,71], also face similar gaps in high-level, longitudinal research evidence and determining feasibility, and methodologies consistent with older adult–driven programs is needed to gather high-quality evidence and offer evidence-based options for both older adults and decision-makers.

Recent work by the Ontario COVID-19 Science Advisory Table demonstrates ways in which NORCs can be better used to support community-dwelling older adults; the Science Table’s members identified NORCs in Toronto, Ontario, Canada, a city of high COVID-19 incidence, for the purpose of rapidly facilitating community-based COVID-19 vaccination clinics [75]; yet, documented examples of using NORCs in such ways are few and far between, likely because of a lack of definition and identification methods. These gaps need to be addressed to better understand both the concept of a NORC and the potential way in which NORCS can be leveraged to support aging adults in the community. To begin with, further work could explore the refinement of the definitions of both NORCs and NORC programs. The literature demonstrated a lack of consensus, especially regarding what constitutes a NORC in terms of size, proportion, age, and other parameters. The literature also lacked methodologies for identifying NORCs. Clarifying both the definition and methodologies would help key stakeholders, including scholars, policy makers, municipalities, and communities, to further identify and describe existing NORCs, adding not only to a growing body of research but also to the growing concept of leveraging NORCs through supportive programs to aid older adults to age in the community.

Some authors of articles included in this review (16/64, 25%) described different engagement activities that sought to empower older adults living in NORCs. Research exploring the village model found that older adults are highly involved in the development of their programs, including policy development, governance, and actual service delivery [76]. Research into connections between engagement and well-being found that older adults who participate in volunteering activities report more positive well-being outcomes [77,78]. Further research should specifically explore the engagement of the older adult participant in NORC programs to examine both the impact of this engagement on older adult well-being and the impact on the success and growth of the program.

Challenges with sustainability, particularly related to funding, have also been reported in research conducted on the village model [79]. Ultimately, this highlights the overall challenge of older adult–driven and community-focused programs to obtain sustainable government-level funding needed to create a stable long-term program. The COVID-19 pandemic has highlighted that most of the government funding for older adults in North America is directed toward institutional care, with much less to home care and even less to community, older adult–driven programs. As a society, we have learned that we need to consider how to better support older adults living in the community to ensure that they can remain safe and connected for as long as possible. This review highlights funding as one of the core requirements for long-term viability.

There was also a surprising lack of information surrounding the question of how much a NORC program costs to run annually. Although variation within different contexts would be expected, this information is critical to present to municipalities or other stakeholders who would be interested in the economic impacts of such a program to determine the budget allocation required to support such programs. In addition, details related to annual costs would help with planning new NORC communities. Further research should explore the cost breakdown of a NORC program and the cost-benefit of the model in comparison with other current means of supporting older adults, including home care and long-term care facilities.

### Conclusions

With our rapidly aging population, there is a clear need to consider how to support older adults living in communities. NORCs hold great promise and are a highly undeveloped approach to developing older adult communities. The body of research around NORCs and NORC programs has been growing for >30 years in North America, and this review provides a critical launching point to begin a focused program of research related to NORCS. NORC programs have the potential to leverage existing resources and partnerships for the purpose of supporting older adults to live well in the community. On the basis of this review, it is clear that further research needs to be conducted to more clearly define what constitutes NORCs and NORC programs, how to identify them in different contexts, and how to create an impact on older adults’ health and well-being over time.

## Abbreviations

NORC: naturally occurring retirement community
NORC-SSP: naturally occurring retirement community supportive service program
PRISMA: Preferred Reporting Items for Systematic Reviews and Meta-Analyses
